# Inflammatory Burden Index (IBI) and Hemoglobin, Albumin, Lymphocyte, and Platelet (HALP) Score in Alzheimer's Disease: A Retrospective Comparative Study

**DOI:** 10.7759/cureus.69148

**Published:** 2024-09-11

**Authors:** Nilifer Gürbüzer, Alev Lazoglu Ozkaya

**Affiliations:** 1 Department of Psychiatry, Erzurum City Hospital, Erzurum, TUR; 2 Department of Biochemistry, Erzurum City Hospital, Erzurum, TUR

**Keywords:** alzheimer's disease, c-reactive protein, hemoglobin albumin lymphocyte and platelet score, inflammation, inflammatory burden index

## Abstract

Objective: This study aimed to evaluate the differences between Alzheimer's disease (AD) patients and controls in biochemistry and peripheral hemogram parameters neutrophil, lymphocyte, monocyte, platelet, and C-reactive protein (CRP) levels, lipid profile, inflammatory burden index (IBI), and hemoglobin, albumin, lymphocyte, and platelet (HALP) score and the relationship between inflammatory and immunonutritive biomarkers and cognitive impairment in patients.

Method: Data from 79 patients with AD and 42 controls were included in the study. Medical data of the participants were obtained from hospital records. IBI was obtained by using the following formula: CRP × neutrophil/lymphocyte. HALP score was calculated as (hemoglobin (g/L) × albumin (g/L) × lymphocytes (/L))/platelets (/L).

Results: Neutrophil count (p=0.003, effect size=0.60), CRP level (p<0.001, effect size=0.87), and IBI (p<0.001, effect size=0.93) were significantly higher in AD patients compared to the control group; hemoglobin (p<0.001, effect size=1.03), lymphocyte count (p<0.001, effect size=0.78), albumin level (p<0.001, effect size=1.31), and HALP score (p<0.001, effect size=0.85) were lower. According to the Standardized Mini Mental Test (SMMT) score, neutrophil count (p=0.001), CRP (p<0.001), and IBI (p<0.001) were significantly higher and lymphocyte count (p=0.001) and HALP score (p<0.001) were lower in the group with severe cognitive impairment. Albumin levels were highest in the group with mild cognitive impairment. In the patient group, there was a moderately significant negative relationship between SMMT score and age (p<0.001, r=-0.437), neutrophil count (p=0.033, r=-0.240), CRP (p<0.001, r=-0.451), and IBI (p<0.001, r=-0.538). Lymphocyte count (p<0.001, r=0.412), high-density lipoprotein (HDL) (p=0.049, r=0.223), albumin levels (p=0.001, r=0.357), and HALP score (p<0.001, r=0.486) were moderately positively associated with SMMT score. Age (β=-0.437, p<0.001), HALP score (β=0.403, p<0.001), and IBI (β=-0.322, p=0.004) were found to be predictors for the severity of cognitive impairment.

Conclusion: Our results revealed that inflammation and immunonutritive status play an important role in the pathogenesis of AD. Novel inflammatory and immunonutritive biomarkers, and IBI and HALP score may be promising clinical tools that may pave the way for more personalized treatment strategies and interventions for patients.

## Introduction

Alzheimer's disease (AD) is the most common form of dementia in the elderly and is also the most common neurodegenerative disorder. It accounts for approximately 60%-70% of all dementia cases. AD causes a slow and progressive decline in both memory and executive cognitive functions [1]. The disease is one of the leading causes of morbidity and mortality in the elderly population and is an important public health problem [2].

Although almost 118 years have passed since the discovery of the disease, its pathogenesis is still poorly understood [3]. Pathologically, AD is characterized by extracellular amyloid beta (Aβ) plaques and intracellular hyperphosphorylated tau neurofibrillary tangles. However, the causal role of these distinctive features in disease development remains unclear. It is clear that many other factors play a role in the onset and progression of AD [4]. Nowadays, there are several hypotheses attempting to explain the early molecular mechanisms of AD pathogenesis. The current pathophysiological approach is based on a number of common mechanisms of neurodegeneration, including abnormal protein (tau and Aβ) accumulation, mitochondrial dysfunction, oxidative stress, impaired insulin signaling, imbalance of neurotransmitters, late apoptotic cell death, dysfunction of microglia, brain-resident macrophages, and neuroinflammation [5].

Recently, the inflammatory hypothesis has become increasingly important in the pathogenesis of AD. Although the relationship between AD and neuroinflammation was discovered a long time ago, it is still not clear whether it is a cause or a consequence of the disease. Acute inflammation in the brain is a well-known defense against infection, toxins, and injury, but when a disruption in the balance of anti-inflammatory and pro-inflammatory signaling occurs, as seen in AD, it results in chronic inflammation (neuroinflammation). Persistent over-activation of pro-inflammatory responses is implicated in the pathogenesis of many neurodegenerative disorders, including AD [4,6].

It is reported in the literature that chronic systemic inflammation and immune activation are associated with AD pathogenesis and that neuroinflammation mediated by astrocytes and glial cells occurs in the brain before the onset of cognitive impairment [7]. It has been reported that the levels of peripheral inflammatory markers such as monocytes and neutrophils are increased in AD, the number of lymphocytes is low, and increased monocyte and neutrophil levels are the hallmarks of chronic inflammation and may be the precursor of AD [8]. C-reactive protein (CRP), which plays a role in the inflammatory response produced by the liver, is used as a biomarker of systemic inflammation in clinical practice [9]. Elevated CRP levels have been reported in AD [10]. In addition to changes in peripheral blood parameters, low hemoglobin and anemia have also been associated with cognitive impairment and AD [11]. It has been reported that anemia has a prevalence of more than 10% in the elderly, increases with age, and is an independent risk factor for cognitive impairment and dementia incidence [12].

The biochemical effects of albumin, which constitutes 50%-60% of plasma proteins, are very diverse. It is the main determinant of plasma oncotic pressure. It is also involved in capillary permeability, hemostasis, immune modulation, anti-inflammatory activity, and endothelial stabilization. It is an important transport protein for therapeutic and toxic substances. Albumin is also the most abundant protein in cerebrospinal fluid [13]. Albumin has several properties of clinical importance for the treatment of patients with AD. Its binding affinity to Aβ peptide and its capacity to inhibit Aβ fibrillization are reported in the literature [13].

The role of lipids in the pathogenesis of AD is controversial. The brain contains approximately 30% of total body cholesterol and is therefore the most cholesterol-rich organ in the body [14]. Given that cholesterol is an essential component of cell membranes and plays an important role in the development and maintenance of neuronal plasticity [14], it can be suggested that lipids may play a direct role in the basic pathological changes in AD. Data from research including epidemiological, animal, cell biology, and genetic studies support a possible role for dyslipidemia and low high-density lipoprotein (HDL) cholesterol levels in the pathogenesis of dementia and AD [14].

In light of the above information, considering the role of platelets, white blood cells and their subtypes, lipids, CRP, and albumin in neuroinflammation, neurodegeneration, and cognitive decline, it can be said that the presence of these parameters outside the brain makes them an accessible and promising therapeutic target for AD.

Inflammatory burden index (IBI) is a new indicator of systemic inflammation and immune status, formulated as CRP × neutrophil/lymphocyte [15]. It was first proposed by Xie et al. as a tool to assess the inflammatory burden of different cancers and to predict the prognosis of patients [16]. The hemoglobin, albumin, lymphocyte, and platelet (HALP) score is a simple and useful indicator reflecting the combination of inflammation and nutritional status, requiring a complete blood count and albumin value. The HALP score is calculated as HALP score = (hemoglobin (g/L) × albumin (g/L) × lymphocytes (/L))/platelets (/L). It was developed by Chen et al. to predict prognosis in gastric carcinoma [17].

In the last few years, although IBI and HALP score have been included in the literature as prognostic biomarkers in various diseases [15-20], studies investigating their relationship with neuropsychiatric diseases [21] are limited. In our study, we investigated the relationship between IBI and HALP score and possible AD to eliminate the research gap and contribute to the elucidation of the pathogenesis of AD. For this, we combined inflammation and nutritional indicators. By evaluating biochemistry and peripheral hemogram parameters hemoglobin, albumin, neutrophil, lymphocyte, monocyte, platelet, and CRP levels, lipid profile, and IBI and HALP score together, we aimed to reveal inflammatory and immunonutritive markers and the relationship between these markers and AD in probable AD. These biomarkers may provide valuable information about the distinctive pathophysiological mechanisms underlying AD. The identification of reliable biomarkers may pave the way for more personalized treatment strategies and interventions and improve the quality of life of individuals with AD and their families.

In light of all this information, the research questions of this study are as follows. (a) Is there a difference between the biochemistry and peripheral hemogram parameters hemoglobin, albumin, neutrophil, lymphocyte, neutrophil, lymphocyte, monocyte, platelet, and CRP levels, lipid profile, and IBI and HALP score of individuals with probable AD and controls? (b) Is there a relationship between the severity of cognitive impairment, biochemistry and peripheral hemogram parameters, and inflammatory and immunonutritive markers? (c) What is the relationship between the new inflammatory and immunonutritive markers IBI and HALP score and the severity of cognitive impairment, and what benefits can they provide in clinical practice?

## Materials and methods

Research design

In this study, a retrospective cross-sectional research design was used. Patients who were admitted to the psychiatry outpatient clinic of Erzurum City Hospital between January 1, 2023, and May 31, 2024, and diagnosed with probable Alzheimer's disease according to history, psychiatric and neurological examination, Diagnostic and Statistical Manual of Mental Disorders, Fifth Edition (DSM-5) diagnostic criteria, and diagnostic criteria determined by the National Institute on Aging-Alzheimer's Association (NIA-AA) working groups were included in the study [22-24]. Secondary causes of dementia were excluded by blood samples and cranial magnetic resonance imaging. The control group consisted of participants of the same age and gender as the patient group, whose cognitive test results did not meet the criteria for dementia, who did not have any comorbidities or regular medication use, and who were examined in the psychiatry outpatient clinic for other reasons such as issuing a medical report and who had no psychopathology. The research protocol of the study was approved by the Health Sciences University Erzurum Medical Faculty Scientific Research Ethics Committee (Erzurum, Turkey) with decision number BAEK 2024/07-143 and was conducted in accordance with the Declaration of Helsinki.

Research sample

In our study, criterion sampling, one of the purposive sampling methods, was used. In this type of sampling, the sample that meets certain predetermined criteria is taken as the basis. The criteria determined by the researcher to explain the situations examined can be used for this type of sampling. In criterion sampling, the individuals planned to be included in the study are determined according to certain criteria [25,26]. The criterion or criteria used can be created by the researcher, or a previously prepared list of criteria can be used [27].

The criteria based on the sample creation for the patient group are as follows: applying to the psychiatric outpatient clinic within the specified date (January 1, 2023-May 31, 2024), being diagnosed with probable AD, not receiving antidementia treatment, being in the age range of 65-90 years, no obesity (body mass index (BMI) < 30 kg/m^2^), not having a history of major psychiatric disorder that started before the onset of neurocognitive disorder, not having comorbid neurological and systemic diseases, and not having a history of infection and immunosuppressive, anticoagulant, or anti-inflammatory drug use in the last two weeks. Additionally, the criteria included having undergone the Standardized Mini Mental Test (SMMT), scoring less than 24 points on SMMT, and having a complete examination of blood parameters.

Patient group data were obtained from 79 patients who were diagnosed with probable AD and met the criteria for the patient group. Of the 146 patients diagnosed with probable AD between January 1, 2023, and May 31, 2024, 12 patients were excluded from the study because they had a previous diagnosis of major psychiatric disorder (such as bipolar disorder, schizophrenia, and major depression). Of the remaining 134 patients, 35 were excluded due to the presence of neurological and systemic diseases (such as stroke, epilepsy, heart disease, hypertension, diabetes, lung and kidney disease, and malignancy), and 20 were excluded due to missing hemogram and biochemical data. As a result, the patient group was formed from the data of a total of 79 patients who fulfilled the inclusion and exclusion criteria.

For the control group, the criteria taken as a basis for the creation of the sample were as follows: being admitted to the psychiatry outpatient clinic within the specified date range (January 1, 2023-May 31, 2024), having no current psychiatric disorder, having no history of major psychiatric disorder and no psychotropic use, age between 65 and 90 years, no obesity (BMI < 30 kg/m^2^), no neurological and systemic diseases, no history of infection, and no history of immunosuppressive, anticoagulant, or anti-inflammatory drug use in the last two weeks. Additionally, the criteria included having undergone the SMMT, scoring 24 points or more on the SMMT, and having blood parameters fully examined.

Control group data were obtained from 42 participants of the same age and gender as the patient group who met the criteria for the control group. Between January 1, 2023, and May 31, 2024, 18 of the 163 participants aged 65-90 years who applied to the psychiatry outpatient clinic for other reasons such as issuing a health report were excluded from the study because they had a previous diagnosis of major psychiatric disorder (such as bipolar disorder, schizophrenia, and major depression). Of the remaining 145 participants, 69 were excluded due to the presence of neurological and systemic diseases (stroke, epilepsy, heart disease, hypertension, diabetes, lung and kidney disease, malignancy, etc.), 26 participants were excluded due to missing hemogram and biochemical data, and eight participants were excluded due to lack of SMMT results. As a result, the control group was formed from the data of a total of 42 participants who met the inclusion and exclusion criteria.

Inclusion and exclusion criteria

The age range of the participants was 65-90 years. There were no gender restrictions among the participants. The exclusion criteria for patients were as follows: obesity (BMI > 30 kg/m^2^), a history of major psychiatric disorders (such as major depression, bipolar disorder, and schizophrenia), presence of comorbid neurological and systemic diseases (cerebrovascular events, Parkinson's disease, epilepsy, heart disease, pulmonary or renal failure, hematological disease, malignancy, connective tissue disease, and acute/chronic inflammatory or autoimmune disease), a history of infection in the last two weeks, a history of immunosuppressive, anticoagulant, or anti-inflammatory drug use, and an SMMT score of 24 or higher.

The exclusion criteria for controls were as follows: obesity (BMI > 30 kg/m^2^), presence of current psychiatric disorder, a history of major psychiatric disorder (such as major depression, bipolar disorder, and schizophrenia), presence of neurological and systemic diseases (cerebrovascular events, Parkinson's disease, epilepsy, heart disease, pulmonary or renal insufficiency, hematological disease, malignancy, connective tissue disease, and acute/chronic inflammatory or autoimmune disease), a history of infection, use of immunosuppressive, anticoagulant, or anti-inflammatory drugs in the last two weeks, and an SMMT score below 24.

Clinical and laboratory data of patients and controls were obtained from medical records.

Data collection tools

Sociodemographic and Clinical Data Form

Medical records of all participants were analyzed. Characteristics such as age, height, weight, BMI, and SMMT score were obtained from hospital records.

Standardized Mini Mental Test (SMMT)

It is a short and useful screening test developed by Folstein et al. (1975) aiming to assess general cognitive functions and administered by the interviewer [28]. The scale consists of 11 items under five main headings: orientation, recording memory, recall, attention and calculation, and language. It is scored between 0 and 30. The Turkish validity and reliability study of the scale was conducted by Güngen et al. (2002) [29]. In the Turkish sample, a cutoff point of 24 points was determined for mild-stage dementia in the SMMT. This cutoff point was found to have a very high sensitivity (0.91) and specificity (0.95) in the diagnosis of mild dementia [29]. According to the SMMT, those scoring below 24 points were considered as probable AD. Those scoring between 23 and 20 points were categorized as mild, 19 and 10 points as moderate, and 0 and 9 points as severe.

Biochemistry and Complete Blood Count Measurement

All blood analyses were performed at Erzurum City Hospital Biochemistry Central Laboratory using an automatic hematological analyzer (Sysmex XN-1000, Sysmex, Kobe, Japan) and a biochemistry (Atellica, Siemens Healthineers, Erlangen, Germany) analyzer.

Normal Reference Ranges for Biochemistry and Hemogram

The normal reference ranges were as follows: neutrophils, 1.8-6.98×10^9^/L: lymphocytes, 1.21-3.77×10^9^/L; monocytes, 0.29-0.95×10^9^/L; platelets, 152-383×10^9^/L: hemoglobin, 14.1-17.8 gr/dL; HDL cholesterol, 35-55 mg/dL; low-density lipoprotein (LDL) cholesterol, 100-129 mg/dL; triglycerides, 0-200 mg/dL; total cholesterol, 0-200 mg/dL; albumin, 32-48 g/L; and CRP, 0-5 mg/L.

IBI was obtained using the following formula: CRP × neutrophils/lymphocytes. HALP score was calculated as follows: (hemoglobin (g/L) × albumin (g/L) × lymphocytes (/L))/platelets (/L).

Statistical analysis

The analyses of the study were performed using IBM Statistical Package for the Social Sciences (SPSS) version 22 (IBM SPSS Statistics, Armonk, NY). The normal distribution of continuous variables was analyzed using the Shapiro-Wilk test, Kolmogorov-Smirnov test, Q-Q plot, skewness, and kurtosis. Data were presented as mean, standard deviation (SD), percentage, and number. Comparisons between categorical variables were made using the Chi-square test. For comparisons between two independent groups, an independent samples t-test was used since the normal distribution condition was met. The one-way analysis of variance (ANOVA) test was used for comparisons of continuous variables with more than two independent groups since the normal distribution condition was met. Post hoc tests after the ANOVA test were performed using the Bonferroni test when variances were homogenous and Tamhane's T2 test when variances were not homogenous. Pearson correlation analysis was used to evaluate the relationship between quantitative variables, and hierarchical multiple regression analysis was used to determine the factors predicting the dependent variable. The statistical significance level was taken as p<0.05.

## Results

Data from 121 participants aged between 65 and 90 years were included in our study. The patient group consisted of 79 participants, 50 of whom were female (63.3%) with probable AD, and the control group consisted of 42 healthy controls, 26 of whom were female (61.9%). The patient and control groups were similar in terms of age, BMI, and gender. The SMMT score of the patients was 14.72±4.99. Neutrophils (p=0.003, effect size=0.60), CRP (p<0.001, effect size=0.87), and IBI (p<0.001, effect size=0.93) were significantly higher in patients compared to controls. Hemoglobin (p<0.001, effect size=1.03), lymphocyte (p<0.001, effect size=0.78), albumin (p<0.001, effect size=1.31) levels, and HALP score (p<0.001, effect size=0.85) of patients were significantly lower than controls (Table 1).

**Table 1:** Comparison of clinical and blood parameters between patient and control groups Note: p<0.05 (statistical significance level in comparison of groups) Effect size=Cohen's d (0.2: small, 0.5: medium, and 0.8: large effect size) BMI: body mass index, CRP: C-reactive protein, HALP score: hemoglobin, albumin, lymphocyte, and platelet score, HDL: high-density lipoprotein, IBI: inflammatory burden index, LDL: low-density lipoprotein, SD: standard deviation, SMMT: Standardized Mini Mental Test, n: number of participants, t: independent samples t-test

Parameters	Patient group (n=79)	Control group (n=42)	t	Effect size	p
	Mean±SD	Mean±SD			
Age (year)	79.72±6.95	77.40±7.34	-1.713	-	0.089
BMI (kg/m^2^)	25.13±3.09	25.39±2.71	0.660	-	0.654
SMMT	14.72±4.99	25.79±1.76	17.734	2.95	<0.001
Hemoglobin (g/dL)	13.31±1.69	14.86±1.28	5.194	1.03	<0.001
Neutrophil count (×10^9^/L)	4.71±1.55	3.85±1.30	-3.075	0.60	0.003
Lymphocyte count (×10^9^/L)	1.82±0.80	2.35±0.53	4.270	0.78	<0.001
Monocyte count (×10^9^/L)	0.57±0.21	0.54±0.12	-0.835	-	0.405
Platelet count (×10^9^/L)	245.33±55.71	279.26±52.63	3.250	0.62	0.001
Triglyceride (mg/dL)	131.65±54.91	134.36±55.08	0.258	-	0.797
HDL cholesterol (mg/dL)	40.84±11.03	45.27±7.81	2.562	0.46	0.012
LDL cholesterol (mg/dL)	116.21±36.59	133.62±40.63	2.398	0.45	0.018
Total cholesterol (mg/dL)	167.46±43.73	195.31±40.09	3.431	0.66	0.001
Albumin (g/L)	38.35±5.92	44.29±2.44	7.759	1.31	<0.001
CRP (mg/L)	6.64±5.56	3.05±1.55	-5.358	0.87	<0.001
IBI	26.17±31.65	5.10±2.96	-5.869	0.93	<0.001
HALP score	40.29±20.99	57.54±19.41	4.413	0.85	<0.001

Lymphocyte count (p=0.027), platelet count (p=0.015), HDL level (p<0.001), LDL level (p=0.001), cholesterol level (p<0.001), and albumin level (p=0.016) were significantly lower in males compared to females in the patient group. According to the SMMT score in patients with probable AD, those who scored between 23 and 20 points were classified as mild cognitive impairment, those who scored between 19 and 10 points were classified as moderate cognitive impairment, and those who scored between 0 and 9 points were classified as severe cognitive impairment. Neutrophil count (p=0.001) was significantly higher and lymphocyte count (p=0.001) was significantly lower in the group with severe cognitive impairment compared to the other two groups. Albumin levels were highest in the group with mild cognitive impairment (p=0.024) compared to the other two groups. CRP (p<0.001), IBI (p<0.001) and HALP score (p<0.001) were significantly different between the three groups. CRP and IBI were highest in the group with severe cognitive impairment, and the HALP score was highest in the group with mild cognitive impairment (Table 2).

**Table 2:** Comparison of clinical and blood parameters of patients with mild, moderate, and severe cognitive impairment Note: p<0.05: statistical significance level in comparison of groups ^abc^Significance between three groups BMI: body mass index, CRP: C-reactive protein, F: one-way ANOVA test value, ANOVA: analysis of variance, HALP score: hemoglobin, albumin, lymphocyte, and platelet score, HDL: high-density lipoprotein, IBI: inflammatory burden index, LDL: low-density lipoprotein, SD: standard deviation, n: number of participants

Parameters	Mild cognitive impairment (n=18)	Moderate cognitive impairment (n=46)	Severe cognitive impairment (n=15)	F	p
	Mean±SD	Mean±SD	Mean±SD		
Age (year)	76.44±6.22^a^	79.93±6.85^abc^	83.00±6.75^c^	3.975	0.023
BMI (kg/m^2^)	24.46±3.04	25.39±3.06	25.14±3.32	0.586	0.559
Hemoglobin (g/dL)	13.54±1.64	13.41±1.65	12.72±1.87	1.163	0.318
Neutrophil count (×10^9^/L)	4.26±1.19^ab^	4.48±1.56^ab^	5.95±1.29^c^	7.102	0.001
Lymphocyte count (×10^9^/L)	2.20±0.98^ab^	1.87±0.69^ab^	1.21±0.57^c^	7.477	0.001
Monocyte count (×10^9^/L)	0.58±0.18	0.57±0.24	0.54±0.18	0.194	0.824
Platelet count (×10^9^/L)	232.33±55.20	247.65±50.09	253.80±72.24	0.698	0.501
Triglyceride (mg/dL)	134.44±56.49	135.41±56.56	116.73±48.44	0.679	0.510
HDL cholesterol (mg/dL)	43.68±12.32	40.99±10.71	36.97±9.91	1.546	0.220
LDL cholesterol (mg/dL)	120.67±32.29	119.00±40.26	102.27±26.91	1.369	0.260
Total cholesterol (mg/dL)	167.39±47.88	17.32±41.94	146.47±39.87	2.376	0.100
Albumin (g/L)	41.61±4.43^a^	37.57±5.75^b^	36.83±6.84^bc^	3.897	0.024
CRP (mg/L)	3.44±3.32^a^	6.49±5.43^b^	10.97±5.57^c^	9.093	<0.001
IBI	7.16±5.67^a^	22.05±28.01^b^	61.62±33.94^c^	19.108	<0.001
HALP score	53.73±21.56^a^	40.44±19.47^b^	23.73±12.14^c^	10.363	<0.001

A moderately significant positive correlation was found between age and CRP (p=0.003), while a moderately significant negative correlation was found between age and albumin levels (p=0.001). In the patient group, a moderately significant negative correlation was found between SMMT score and age (p<0.001), neutrophil count (p=0.033), CRP (p<0.001), and IBI (p<0.001). A moderately significant positive correlation was found between SMMT score and lymphocyte count (p<0.001), HDL (p=0.049), albumin levels (p=0.001), and HALP score (p<0.001) (Table 3).

**Table 3:** Correlation of clinical and blood parameters in the patient group Note: p<0.05: statistical significance level BMI: body mass index, CRP: C-reactive protein, HALP score: hemoglobin, albumin, lymphocyte, and platelet score, HDL: high-density lipoprotein, IBI: inflammatory burden index, LDL: low-density lipoprotein, SMMT: Standardized Mini Mental Test

Parameters	1	2	3	4	5	6	7	8	9	10	11	12	13	14	15	16
Age	1	-	-	-	-	-	-	-	-	-	-	-	-	-	-	-
	-	-	-	-	-	-	-	-	-	-	-	-	-	-	-	-
BMI	-0.052	1	-	-	-	-	-	-	-	-	-	-	-	-	-	-
	0.648	-	-	-	-	-	-	-	-	-	-	-	-	-	-	-
SMMT	-0.437	-0.078	1	-	-	-	-	-	-	-	-	-	-	-	-	-
	0.000	0.495	-	-	-	-	-	-	-	-	-	-	-	-	-	-
Hemoglobin	-0.168	0.056	0.180	1	-	-	-	-	-	-	-	-	-	-	-	-
	0.138	0.622	0.112	-	-	-	-	-	-	-	-	-	-	-	-	-
Neutrophil	-0.031	-0.007	-0.240	-0.025	1	-	-	-	-	-	-	-	-	-	-	-
	0.786	0.954	0.033	0.828	-	-	-	-	-	-	-	-	-	-	-	-
Lymphocyte	-0.186	0.050	0.412	0.288	-0.048	1	-	-	-	-	-	-	-	-	-	-
	0.102	0.663	0.000	0.010	0.672	-	-	-	-	-	-	-	-	-	-	-
Monocyte	0.059	-0.042	0.061	0.230	0.246	0.312	1	-	-	-	-	-	-	-	-	-
	0.604	0.712	0.590	0.041	0.029	0.005	-	-	-	-	-	-	-	-	-	-
Platelet	-0.061	-0.040	-0.098	-0.045	0.242	2.272	0.075	1	-	-	-	-	-	-	-	-
	0.592	0.724	0.390	0.693	0.032	0.015	0.510	-	-	-	-	-	-	-	-	-
CRP	0.330	0.122	-0.451	-0.269	0.397	-0.291	0.062	-0.034	1	-	-	-	-	-	-	-
	0.003	0.286	0.000	0.017	0.000	0.009	0.586	0.768	-	-	-	-	-	-	-	-
Triglyceride	-0.127	-0.075	0.130	0.023	0.065	0.469	0.071	0.246	-0.206	1		-	-	-	-	-
	0.263	0.510	0.254	0.838	0.569	0.000	0.533	0.029	0.068	-	-	-	-	-	-	-
HDL	-0.121	0.030	0.223	0.164	-0.217	0.293	-0.055	0.068	-0.397	-0.114	1	-	-	-	-	-
	0.288	0.796	0.049	0.148	0.055	0.009	0.632	0.553	0.000	0.318	-	-	-	-	-	-
LDL	0.072	0.142	0.146	0.152	-0.242	0.279	-0.120	0.035	-0.200	0.489	0.276	1	-	-	-	-
	0.529	0.213	0.198	0.181	0.032	0.013	0.290	0.757	0.078	0.000	0.014	-	-	-	-	-
Cholesterol	0.013	0.046	0.136	0.143	-0.195	0.337	-0.134	0.063	-0.273	0.453	0.515	0.779	1	-	-	-
	0.907	0.690	0.232	0.209	0.085	0.002	0.238	0.584	0.015	0.000	0.000	0.000	-	-	-	-
Albumin	-0.361	-0.156	0.357	0.320	0.032	0.266	0.147	0.034	-0.500	0.143	0.353	-0.017	0.169	1	-	-
	0.001	0.171	0.001	0.004	0.779	0.018	0.195	0.765	0.000	0.208	0.001	0.882	0.137	-	-	-
IBI	0.302	0.129	-0.538	-0.393	0.517	-0.519	-0.035	-0.055	0.847	-0.233	-0.412	-0.270	-0.343	-0.453	1	-
	0.007	0.258	0.000	0.000	0.000	0.000	0.759	0.629	0.000	0.039	0.000	0.016	0.002	0.000	-	-
HALP score	-0.249	0.079	0.486	0.535	-0.126	0.802	0.249	-0.199	-0.334	0.321	0.290	0.236	0.303	0.465	-0.539	1
	0.027	0.489	0.000	0.000	0.269	0.000	0.027	0.079	0.003	0.004	0.010	0.036	0.007	0.000	0.000	-

A hierarchical multiple regression analysis was performed to determine the factors predicting cognitive impairment (according to the SMMT result) in people with probable AD. The models were tested by taking the previously determined independent variables in a determined order. In the first step, the age of the patients, in the second step the HALP score, and in the third step the IBI independent variables were included in the model. The results of the analyses related to the evaluation of the model are given in Table 4. When the analysis results are analyzed, it is seen that the age variable in the first model explains 19.1% of the SMMT score. In the second model, 34.3% of the variance is explained with the HALP score included later. The variance explained in the second model is the variance explained by all independent variables (age and HALP score). To see how much of the overall variance was explained by the HALP score, the R2 change value was examined on the second model, and it was found that this value was 0.152. This value means that when the age variable is controlled, the HALP variable explains 15.2% of the variance in the SMMT score (R2 change=15.2, p=0.000). In the third model, 41.4% of the variance is explained with the IBI included later. The variance explained in the third model is the variance explained by all independent variables (age, HALP score, and IBI). To see how much of the overall variance is explained by IBI, the R2 change value was examined on the second model, and it was found that this value was 0.071. This value means that when age and HALP score variables are controlled, the IBI variable explains 7.1% of the variance on the SMMT score (R2 change=7.1, p=0.004).

**Table 4:** Analysis results regarding the evaluation of the model Note: p<0.05: statistical significance level ^a^Predictor: (Constant), age ^b^Predictor: (Constant), age, HALP score ^c^Predictor: (Constant), age, HALP score, IBI Dependent variable: SMMT HALP score: hemoglobin, albumin, lymphocyte, and platelet score, IBI: inflammatory burden index, SMMT: Standardized Mini Mental Test

Model	R	R^2^	Adjusted R^2^	Standard error	Change statistics				
					R^2 ^change	F change	df1	df2	Sig. F change
1	0.437^a^	0.191	0.180	4.519	0.191	18.167	1	77	0.000
2	0.589^b^	0.343	0.320	4.099	0.152	17.597	1	76	0.000
3	0.650^c^	0.414	0.390	3.898	0.071	9.017	1	75	0.004

The results of the hierarchical multiple regression analysis are given in Table 5. When the independent variable in the first model is analyzed, it is seen that the age variable has a predictive effect on the SMMT score (β=-0.437, p<0.001). This model can be expressed as "SMMT score=39.75+(-0.437*age)." According to this model, it can be interpreted that a one-unit change in age causes a negative change of 0.437 units on the SMMT score. In other words, as age increases, SMMT score decreases. In the second model, age (β=-0.337, p=0.001) and HALP score (β=0.403, p<0.001) have a prediction effect on SMMT score. In the third model, age (β=-0.279, p=0.004), HALP score (β=0.243, p=0.024), and IBI (β=-0.322, p=0.004) had a predictive effect on SMMT score. The results can be interpreted as a decrease in SMMT score as age and IBI increase and an increase in SMMT score as HALP score increases.

**Table 5:** Hierarchical multiple regression analysis results Note: p<0.05: statistical significance level HALP score: hemoglobin, albumin, lymphocyte, and platelet score, IBI: inflammatory burden index, SMMT: Standardized Mini Mental Test (dependent variable)

Model		Unstandardized coefficients		Standardized coefficients	t	p
		B	Standard error	Beta		
1	(Constant)	39.747	5.893	-	6.744	0.000
	Age	-0.314	0.074	-0.437	-4.262	0.000
2	(Constant)	30.150	5.814	-	5.186	0.000
	Age	-0.242	0.069	-0.337	-3.508	0.001
	HALP score	0.096	0.023	0.403	4.195	0.000
3	(Constant)	29.713	5.532	-	5.371	0.000
	Age	-0.1201	0.067	-0.279	-2.993	0.004
	HALP score	0.058	0.025	0.243	2.303	0.024
	IBI	-0.051	0.017	-0.322	-3.003	0.004

## Discussion

This study evaluated the differences in biochemistry and peripheral hemogram parameters neutrophil, lymphocyte, monocyte, platelet, and CRP levels, lipid profile, IBI and HALP score, and the relationship between inflammatory and immunonutritive markers and cognitive impairment in patients with possible AD and healthy controls. Our results revealed that compared with healthy controls, AD patients had poor nutritional status, systemic inflammation occurred, and age, IBI, and HALP scores were predictive of the severity of cognitive impairment.

In our study, it was found that neutrophil count was increased, lymphocyte count was decreased, CRP was high, and systemic inflammation occurred in patients with AD in accordance with the literature [8,10,30,31]. In a study including 27 patients with AD, neutrophil count was found to be high and lymphocyte count was found to be low, and it was suggested that white blood cells may be peripheral markers for early diagnosis of the disease together with other inflammatory markers [8]. In a recent review including AD mouse models and human cohort studies, neutrophils were reported to potentially contribute to increased neuroinflammation, blood-brain barrier disruption, and cognitive impairment in patients [7]. It has been reported that peripheral inflammation in AD may increase blood-brain barrier permeability, causing some vasculotoxic and neurotoxic proteins to enter the brain, increased extravasation of plasma proteins in the brain parenchyma may activate microglia and astrocytes, and blood-brain barrier dysfunction may also contribute to decreased Aβ clearance [10]. It has even been suggested that people using non-steroidal anti-inflammatory drugs (NSAIDs) may be protected from AD, the incidence of AD is lower in patients with rheumatoid arthritis receiving long-term NSAIDs treatment, and the longer NSAIDs are used before clinical diagnosis, the greater the protective effect [2].

IBI, which is calculated as CRP × neutrophil/lymphocyte, was found to be higher in our study compared to controls. IBI is a new systemic inflammation biomarker developed by Xie et al. to effectively predict the prognosis of cancer patients in a study involving 6,359 cancer patients with multiple solid tumor types [16]. It has been reported that IBI can be used as a useful predictor of surgical site infection, as well as the prognosis of patients after esophagectomy in patients with esophageal cancer [15]. IBI has been found to be associated with poor prognosis in patients with aneurysmal subarachnoid hemorrhage [32] and acute ischemic stroke [18], except in patients with cancer. No study investigating the association of IBI with AD was found in the literature. In a study including 102 patients with AD, the neutrophil/lymphocyte ratio was found to be high in patients, but this ratio was not found to be associated with the severity of the disease [30]. The IBI obtained by adding CRP to the neutrophil/lymphocyte ratio showed strong correlations with the severity of cognitive impairment in our study. Age is one of the important risk factors for dementia [1]. When the effect of the age variable was controlled, IBI was found to be predictive of the severity of cognitive impairment. It has been reported that IBI is the best inflammatory biomarker among biomarkers associated with systemic inflammation, and the advantage of IBI may be due to the fact that it measures the balance between immune and acute inflammation by combining hematological biomarkers neutrophils, lymphocytes, and CRP [16,18].

In our study, albumin levels, lymphocyte counts, and hemoglobin levels were found to be lower in patients compared to controls. Albumin levels and lymphocyte counts were negatively correlated with the severity of cognitive impairment. Both inflammation and malnutrition risk have been associated with low albumin levels and lymphocyte counts [19,20,33,34]. It is known that albumin has a role in immune modulation, anti-inflammatory activity, and endothelial stabilization [13]. It is suggested that albumin inhibits the formation of Aβ peptide fibrils and supports neuronal survival by preventing the entry of amyloid into neurons, and therefore, low albumin levels in the brain and cerebrospinal fluid may lead to increased Alzheimer-type pathology [13,35]. Studies have shown that low serum albumin levels are associated with an increased likelihood of both dementia and mild cognitive impairment in the elderly population after adjustment for age, gender, education, and additional risk factors for cognitive impairment [35,36]. Albumin and hemoglobin have been identified in a panel of blood-based protein biomarkers to distinguish individuals with AD from cognitively healthy controls [37]. Anemia is common in the elderly population and has been reported to be a risk factor for AD and dementia [11,12]. Although our results are consistent with the literature, reverse causality is also possible. Individuals with cognitive impairment may be malnourished, which may lead to low serum albumin levels and anemia.

Albumin levels, lymphocyte count, and hemoglobin are indicators of nutritional status included in nutritional assessment and screening tools [15,19,20,34]. The HALP score, first developed by Chen et al. to predict gastric carcinoma prognosis, is an immunonutritive biomarker that combines several routinely collected indicators to reflect a patient's general health status in a single composite score [17,19]. In the literature, it is reported that the HALP score can predict the prognosis of patients with cancer in many cancer types other than gastric cancer [19,20]. However, there is limited data on its relationship with neuropsychiatric diseases. It has been reported that a low HALP score is associated with early-onset cognitive impairment after stroke in patients with acute ischemic stroke [21]. In our study, the HALP score was significantly lower in patients with AD compared to controls. In patients, the HALP score was associated with the severity of cognitive impairment, and a low HALP score was predictive of the severity of cognitive impairment. Since anemia, nutritional status, and peripheral inflammation associated with hypoalbuminemia are preventable and treatable, our results are promising for the pathogenesis of the disease.

In our study, HDL, LDL, and cholesterol levels of AD patients were found to be lower than controls. The findings of studies examining the effect of lipids on AD pathogenesis are contradictory in the literature [14]. These results may be due to the age at which cholesterol measurements were performed and the timing in relation to the onset of symptoms. In their study, Notkola et al. reported that high cholesterol was associated with a higher risk of AD after 30 years but that cholesterol levels declined before the onset of AD [38]. In an 18-year longitudinal study of participants over the age of 70, it was shown that high cholesterol present before the onset of dementia was associated with a reduced risk of dementia among 70-year-olds [39]. In a 26-year cohort of men examining the natural history of change in total cholesterol over a period of 10-15 years, those who developed dementia were reported to have a greater decline in cholesterol levels over a period of 10-15 years [40]. A fall in cholesterol levels just before the onset of the disease may be a marker of early processes reflecting neurodegenerative changes and leading to deterioration in general health. Considering the beneficial effects of cholesterol, such as being a precursor of steroid hormones (estrogens, androgens, and vitamin D), providing structural integrity, modulating the fluidity of cell membranes, being an antioxidant, and being necessary for synaptic integrity and neurotransmission, it is possible that it may play a role in protection against dementia [39]. In conclusion, if high cholesterol has a protective role against AD in the elderly, the risk-benefit ratio of lowering cholesterol in this population may need to be re-evaluated.

Our study has strengths and limitations. The retrospective and cross-sectional design of the study is an important limitation. It prevents the establishment of strong causal relationships between blood parameters, biomarkers, and disease-related outcomes. In addition, differences in the time of blood collection and whether blood was collected under appropriate conditions (such as fasting and satiety) may have caused changes in some parameters (such as lipids). Although strict inclusion and exclusion criteria were applied in the study, since the data were obtained from hospital records, treatments and disease diagnoses not included in hospital records may have been overlooked. Another limitation of our study is that although we included patients who did not receive antidementia treatment, we do not know how long their symptoms continued at the time of diagnosis, that is, the duration of the disease, because the systemic inflammatory response and nutritional status may change in relation to the duration of symptoms. The fact that variables such as smoking status and duration, which may contribute to inflammation, were not included in the study is also an important limitation of our study. Another limitation is that the caregivers who would affect the nutritional status were not selected appropriately. For example, the nutritional status of people staying in a nursing home and people cared for by family members may differ. Another limitation of our study is our small sample size due to the design of our study. The SMMT was administered to the participants by experienced psychologists. Administering the SMMT to the participants by different people is both a limitation and an advantage. This may have prevented potential bias. On the other hand, our results revealed that systemic inflammatory response was increased, immunonutritive status was impaired, and inflammatory and immunonutritive markers were associated with the severity of cognitive impairment in AD. Given the role of systemic inflammation and immunonutritive status in the pathogenesis of AD, the novel inflammatory and immunonutritive biomarkers IBI and HALP score may be promising therapeutic targets for AD. Given the limitations of our study, further validation is needed for the clinical relevance of our results. Therefore, prospective follow-up of these groups is important to see the progress in the patient and control groups. Longitudinal and well-designed studies involving larger patient groups should be conducted for the results of the study to be generalizable. In this study, which aims to reflect a real-world sample of the society, it should be kept in mind that our study results may not represent the entire population.

## Conclusions

In this study, it was shown that patients had increased neutrophil counts, decreased lymphocyte counts, increased CRP levels, and increased IBI, which indicates systemic inflammation. Compared to controls, patients had lower levels of albumin, HDL, LDL, cholesterol, and hemoglobin and low HALP scores. The SMMT scores of the patients show a moderate negative correlation with age and inflammatory response. In addition, a moderate positive correlation was found between albumin and HDL levels, lymphocyte count, and the HALP score. IBI and HALP score are associated with the severity of cognitive impairment. The HALP score is low and IBI is high in patients with severe cognitive impairment. When the effect of the age variable was controlled, IBI and HALP score were found to be determinants of the severity of cognitive impairment.
